# Atypical involvement of central nervous system in classic Hodgkin lymphoma: a case report

**DOI:** 10.1186/s13256-021-03118-4

**Published:** 2021-10-29

**Authors:** Shanila Ahmed, Babar Irfan, Muhammad Raza, Ghulam Haider

**Affiliations:** 1 Department of Oncology, Aga Khan University Hospital, Karachi, Pakistan; 2 Internal Medicine Resident Physician, Jinnah Postgraduate Medical Center, Karachi, Pakistan; 3 Department of Pathology and Laboratory Medicine, Aga Khan University Hospital, Karachi, Pakistan; 4 Department of Oncology, Jinnah Postgraduate Medical Center, Karachi, Pakistan

**Keywords:** Central nervous system, Hodgkin lymphoma, Chemotherapy

## Abstract

**Background:**

Hodgkin lymphoma is a systemic disease that commonly involves the cervical, supraclavicular, and mediastinal lymph nodes. The involvement of central nervous system in Hodgkin lymphoma is extremely rare, and diagnosis is usually established using distinct morphological and immunohistochemical staining on the tissue biopsied. Extranodal presentation of HL is a rare occurrence. It has been evident that prognosis is encouraging in patients with disease that is limited to just central nervous system initially or as relapse, compared with involvement of multiple sites of relapse.

**Case presentation:**

We herein report a case of a 35-year-old South-East Asian male with relapsed Hodgkin lymphoma. The patient developed a parotid gland lesion, cervical lymphadenopathy with significant weight loss, and intermittent night sweats. Along with spread to the central nervous system, there was a high suspicion of tuberculosis. Upon biopsy of his cervical lymph node, the patient was confirmed to have Hodgkin lymphoma. Immediate treatment began with six cycles of chemotherapy consisting of adriamycin, bleomycin, vinblastine, and dacarbazine. The patient received three cycles of chemotherapy consisting of ifosfamide, carboplatin, and etoposide but then was lost to follow-up. Five years later, the patient suffered a road traffic accident. Upon work-up, a right parietal space-occupying lesion with moderate cerebral edema and midline shift was found on computed tomography of the brain. The patient underwent resection of the space-occupying lesion of brain, with features consistent with classical Hodgkin lymphoma on histopathology examination. It is crucial for such lesions to be investigated meticulously to rule out any secondary disease process.

**Conclusion:**

Relapsed Hodgkin lymphoma with central nervous system involvement is relatively rare with just over two dozen cases reported to date and is observed infrequently in developing nations. Therefore, space-occupying lesion should always be investigated, and biopsy of such lesions is gold standard to establish diagnosis. With timely appropriate therapy, complete remission can be achieved. However, large-scale studies would be prudent to explore the presentation, survival, and treatment options for patients with Hodgkin lymphoma involving the central nervous system.

## Background

Classic Hodgkin lymphoma (HL) is a cancer of the lymphatic system and primarily affects lymph nodes. As compared with non-Hodgkin lymphoma (NHL), central nervous system (CNS) involvement in HL is extremely rare, recently shown to occur in less than 0.2–0.5% of HL patients [1] whereas CNS involvement can be found in up to 5–30% of NHL patients [2]. Nodular sclerosis and mixed cellularity are the most commonly seen histological subtypes of HL that occur in relapsed disease. Treatment modalities include radiotherapy alone or in combination with chemotherapy. We describe a patient with classic HL presenting with relapse of disease in the brain. The rare occurrence and paucity of available reports on such presentation make it a challenging diagnosis for physicians. Moreover, these presentations can prove to be a puzzling conundrum for physicians in low-middle-income countries where timely diagnosis and management can relieve the existing burden on the under-resourced healthcare.

## Case presentation

A 35-year-old South-East Asian male, nonsmoker, with no known comorbidities, reported to outpatient facility in 2012, when he developed right parotid swelling and was managed conservatively. His swelling later transformed into an ulceration. He took multiple courses of antibiotics prescribed by general physicians and also used medication given by unqualified practitioners . Family history was negative for any malignancies. The patient belonged to a household of low socioeconomic status, being employed as a domestic worker. In a few months’ time, the patient developed progressive cervical lymphadenopathy with significant weight loss and intermittent night sweats. Due to high prevalence of tuberculosis in the local population, the patient underwent antituberculosis treatment (ATT) consisting of rifampicin (10 mg/kg), isoniazid (5 mg/kg), ethambutol (15 mg/kg), and pyrazinamide (25 mg/kg). However, no signs of improvement were observed after treatment for 2 months. During this time period, he developed an ulcerated lesion of the right parotid gland. Biopsy of the cervical lymph node revealed Hodgkin lymphoma, and the patient underwent six cycles of chemotherapy ABVD [adriamycin (25 mg/m^2^ intravenous), bleomycin (10 U/m^2^ intravenous), vinblastine (6 mg/m^2^ intravenous), and dacarbazine (DTIC) (375 mg/m^2^ intravenous)]. Each cycle was repeated every 28 days, and this combination was administered on day 1 and day 15. No significant reduction in size of the lymph nodes was observed after chemotherapy, and his parotid gland lesion remained unchanged. Later in June 2013, a biopsy of the ulcerated parotid gland lesion was performed with findings that were consistent with Hodgkin lymphoma. He received his second-line chemotherapy: three cycles of dexamethasone, high-dose Ara-C, and platinol (DHAP). Staging computed tomography (CT) was performed to evaluate the response of treatment, which showed no interval change in parotid lesion but a significant reduction in the size of cervical lymph nodes. Furthermore, 25 fractions of radiation therapy were administered to the patient at the right side of his face including right side of neck. After 10 months of treatment, disease progression was still observed with complaint of cervical lymphadenopathy. In 2014, the patient received three cycles of chemotherapy consisting of ifosfamide, carboplatin, and etoposide (ICE). The patient demonstrated noncompliance to further treatment options and regular hospital visits because of financial constraints, and was eventually lost to follow-up.

**Fig. 1:**
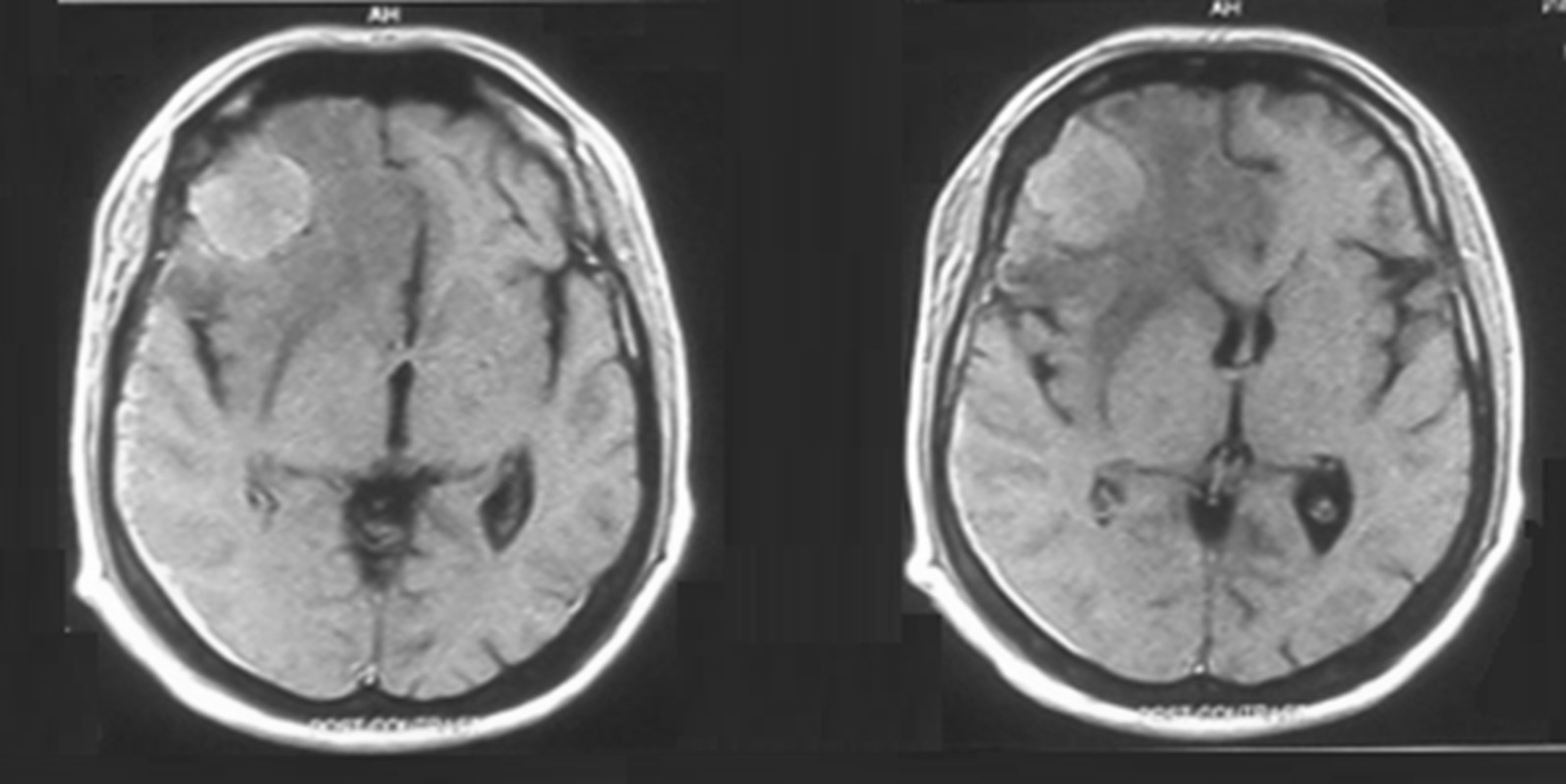
Radiographical presentation showing space-occupying lesion in right parietal region

**Fig. 2:**
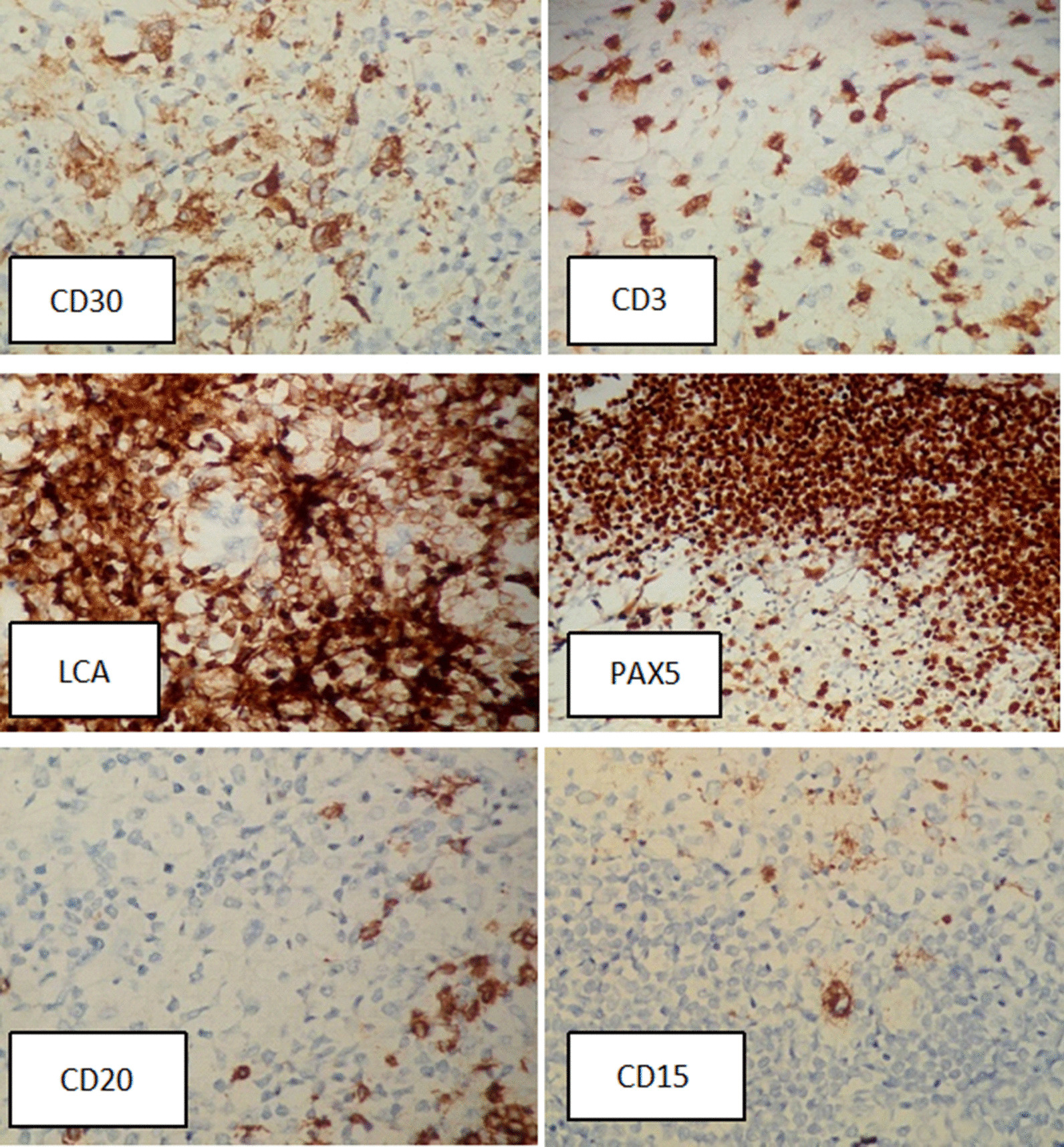
Immunohistochemistry stained tissue biopsy of the lesion showing CD30(+), CD15(+), PAX5(+),CD3(−),CD20(−), and LCA(−)

In 2019, the patient suffered a road traffic accident (RTA) after 5 years of disease. The cause of RTA was identified as loss of consciousness (LOC) as confirmed by the patient’s brother, who was with him during the accident. A right parietal well-circumscribed space-occupying lesion of around 6.7 × 4.9 cm with moderate cerebral edema and midline shift was found on CT brain imaging as shown in Fig. 1. He regained his consciousness spontaneously after a few hours in the emergency department. On further questioning, the patient described intermittent mild headaches a few months prior to the accident. His general physical examination was normal, with few marks of skin abrasions on his limbs. His lab workup was also unremarkable. He was scheduled for elective surgery and eventually underwent a gross total resection of the space-occupying lesion of brain. The specimen was sent for analysis, and immunohistochemistry revealed atypical cell infiltrates largely positive for CD30, CD15, and weakly positive PAX5 but lack of expression for LCA, CD20, and CD3 as shown in Fig. 2. Surprisingly, these features were consistent with classical Hodgkin lymphoma according to the World Health Organization (WHO) classification of lymphoid neoplasm. The patient was reported to be alive and recovering well post-surgery during hospital follow-ups and was also advised to see medical oncology for further cancer treatment. His parotid gland ulceration responded to radiation therapy and very much regressed when he presented with lymphadenopathy for the third time and received three cycles of ICE. Unfortunately, he never showed up and died after 4 months of his surgery, likely due to untreated underlying cancer. Informed consent was obtained from the patient’s uncle for the use of images and details for reporting the case for publication and teaching purposes.

## Discussion

Central nervous system involvement is rare with HL, accounting for 0.2–0.5% of all cases [1], and mostly occurs as a relapse. Due to the rare occurrence, only 22 cases to date have been reported in the literature [1, 3–5]. In contrast, CNS involvement is 5–30% in patients with NHL [2]. Classical HL primarily affects the lymph nodes as neurological sequelae rarely occur. However, neurological complications can occur due to metastatic disease, occurring as paraneoplastic phenomena, or treatment-induced neurological complication [6]. Some of the paraneoplastic manifestations include degeneration of cerebellum, chorea, demyelinating polyradiculopathy, and myasthenia gravis. Therapeutic radiation can lead to neurologic complications such as dropped head syndrome, acute brachial plexopathy, and intracranial infarction. Furthermore, chemotherapy for Hodgkin lymphoma may cause peripheral neuropathy and cerebral infarction possibly due to drug-induced embolism [7].

Most research studies state the location of the lesion to be supratentorial [8]. Several risk factors are responsible for disease relapse such as male gender, positive family history, immunosuppressed state, and infection with Epstein–Barr virus (EBV) [9]. In our case, tuberculosis was suspected since its coexistence with HL is well reported, resulting from two potential processes: T-cell dysfunction related to HL, and B-cell immune dysfunction resulting from HL therapy [10]. Tuberculosis and HL can both involve the CNS, but to date, there is no literature that correlates an increased risk of CNS tuberculosis with HL.

HL can affect CNS in several ways, including skull contiguity, meningeal invasion, or through the hematogenous route, which is the most common route of spread [11]. Some of the common manifestations of intracranial involvement include cranial nerve palsies, with other signs and symptoms such as headaches, papilledema, paresis, seizures, and other neurological manifestations [12]. In our case, the patient had a history of headaches and an episode of loss of consciousness. Nodular sclerosis and mixed cellularity are the most commonly seen histological subtypes of HL that occur in relapsed disease. Moreover, the presence of Reed–Sternberg cells and co-expression of CD30 and CD15 antigens with lack of CD20 and CD3 strongly support the diagnosis of Hodgkin lymphoma [13]. The gold-standard diagnosis is biopsy, which is essential for confirmation.

Due to the rare occurrence of this presentation and insufficient data from clinical trials, no specific treatment guidelines have been established. Evidence suggests a variety of treatment modalities, including radiotherapy alone for isolated CNS involvement, or holocranial irradiation and combination chemotherapy. In the case of meningeal disease, intrathecal methotrexate can be used as well [4]. Other previously used modalities include stem cell transplantation or surgery. CNS prophylaxis is not routinely recommended in HL as more data are needed to identify specific populations who could benefit from it [14]. It is crucial for prompt screening and diagnosis of debilitating complications that have low risk of suspicion and paucity of evidence on treatment. Developing an appropriate treatment strategy has been challenging due to the short median survival of approximately 46 months [6] after HL has been diagnosed. It has been observed that prognosis is better in patients with disease limited to CNS either initially or as relapse, compared with patients with evidence of more than one site of relapse [15].

## Conclusion

Relapsed Hodgkin lymphoma with CNS involvement is uncommon; therefore, space-occupying lesion (SOL) should always be investigated and biopsy of such lesions is gold standard to establish diagnosis. With timely appropriate therapy, complete remission can be achieved. However large-scale studies are still needed to understand the presentation, survival, and treatment options for patients with CNS-HL.

## Data Availability

Not applicable as patient’s information was held by the hospital.
